# Using Resin Infiltration Technique and Direct Composite Restorations for the Treatment of Carious Lesions with Different Depths

**DOI:** 10.1155/2023/5908006

**Published:** 2023-04-15

**Authors:** İsmail Serhat Sadıkoğlu

**Affiliations:** Faculty of Dentistry, Restorative Dentistry Department, European University of Lefke, Lefke, Turkey

## Abstract

Minimally invasive treatment methods are of special interest in restorative dental practice, with numerous methods emerging in the last decade. Such methods are being developed to encompass various applications, an important one being the detection and treatment of caries in the early stages. White spot lesions are the earliest visible stage of the caries process. These lesions have a chalky, opaque appearance, which results in esthetic dissatisfaction. In contrast to the principles of minimally invasive dentistry, considerable amounts of sound tooth structure need to be sacrificed to get rid of these lesions. Therefore, caries infiltration has been introduced as an alternative treatment option for non-cavitated lesions. The resin infiltration technique only works in non-cavitated lesions. Replacement of lost dental tissue with resin composites remains the mainstay therapy in cases with cavity formation. This case report describes a case of caries with lesions of varying depths. In such cases, a combination of treatment methods may be used to provide satisfying esthetics with a minimally invasive approach.

## 1. Introduction

Dental caries is the most common oral disease in the world [1]. Initial caries lesions—known as white spot lesions—are the first visible indicator of enamel caries, which can appear as early as two weeks after plaque accumulation [2]. Initial caries lesions can be treated with invasive or non-invasive treatment options. Invasive treatment options, such as porcelain laminate veneers, can be used for the esthetic management of lesions that do not respond adequately to non-invasive approaches [3]. However, these methods are generally associated with excessive tissue loss. The non-invasive treatment options are conservative approaches that aim to remineralize the initial caries lesions [4]. However, multiple clinical studies demonstrated that these remineralization processes do not produce cosmetic improvements, as evaluated by the International Caries Detection and Assessment System criteria [5]. Apart from all these, the resin infiltration method, which is classified as a micro-invasive approach, has proven its success in various clinical and laboratory studies [6–8]. The idea of resin infiltration is based on the use of low-viscosity resins to fill the porous structure of the initial enamel carious lesions, which have an intact surface layer [9, 10]. However, in cases where the depth of the lesion increases and a cavity formation occurs, resin infiltration alone is insufficient, and the cavity must be restored with composite resins. As presented in this case report, sometimes lesions of different depths forms together. In this case, it would be wise to use a combination of different restorative techniques. The report is of clinical significance as it demonstrates the advantages of combining two different approaches in one patient, which are micro-invasive and minimally invasive approaches.

## 2. Case Presentation

**Figure 1:**
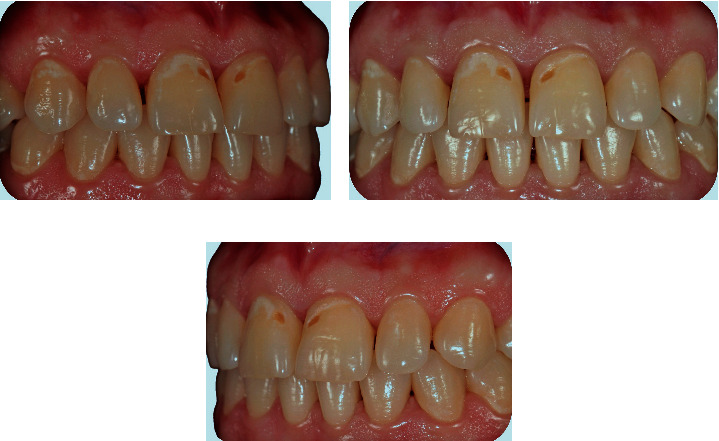
Initial views of caries lesions. (a) Right angle, (b) Front angle, (c) Left angle.

A 32-year-old woman applied to our clinic with esthetic complaints caused by caries lesions on her anterior teeth after pregnancy. In the clinical intraoral examination, caries lesions of various depths, which were concentrated around the gingival regions were detected in the upper anterior teeth (Figure 1). Turesky modification of the Quigley and Hein plaque index was used to record plaque index, and the score was recorded as 2. The patient's story revealed frequent reflux and vomiting attacks that were experienced during pregnancy. In addition, the patient stated that she had stopped brushing to avoid the associated emetic effects of brushing. After discussing the treatment options and esthetic expectations with the patient, we agreed on treating the lesions at the enamel level by the resin infiltration method and the cavitated lesions by direct composite restorations. Before the treatment stage, signed informed consent was obtained.

**Figure 2:**
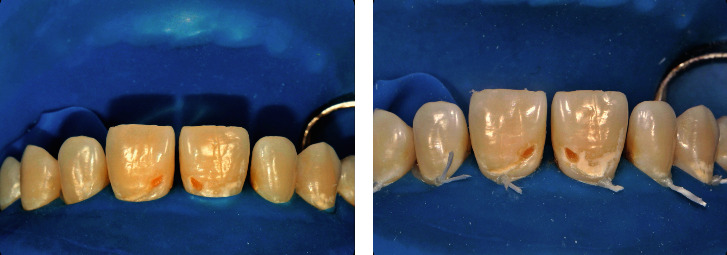
(a) Rubber dam isolation. (b) Addition of floss tie knots to ensure soft tissue retraction.

In the first session, periodontal prophylaxis was applied, and oral hygiene instructions were given. The second appointment was set for a week later to allow time for the patient to restore her habits of oral hygiene. In the second appointment, the plaque index score was measured as 0. Considering the risk of dehydration, the appropriate color was selected with the composite button technique before any procedure was performed. After the selection of color, a rubber dam was applied for isolation (Figure 2(a)). A floss tie was used to ensure soft tissue retraction and rubber dam inversion, revealing the margins of the lesions (Figure 2(b)).

### 2.1. First Treatment Step: Resin Infiltration of the Superficial Enamel Lesions

**Figure 3:**
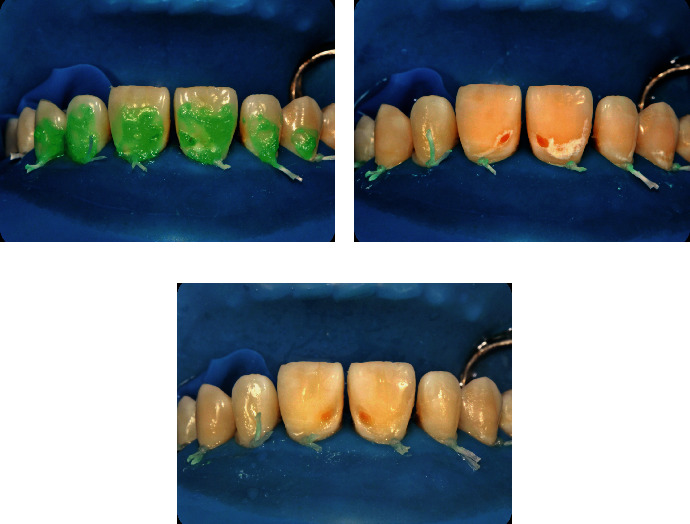
Resin infiltration technique steps: application of (a) Icon-Etch, (b) Icon-Dry, and (c) Icon-Infiltrant.

The resin infiltration technique was applied for the treatment of non-cavitated lesions as follows. The enamel surfaces were first etched by the application of 15% hydrochloric acid gel (Icon-Etch, DMG, Hamburg, Germany) for 2 minutes followed by a water rinse for 30 seconds (Figure 3(a)). The lesions were then dried with ethanol (Icon-Dry, DMG) for 30 seconds (Figure 3(b)). Subsequently, a low-viscosity resin infiltrant (Icon-Infiltrant, DMG) was applied to the lesions for 3 minutes, which were then light-cured for 40 seconds with a Light Emitting Diode (LED) curing device (Elipar S10, 3M ESPE, St. Paul, MN, USA; Figure 3(c)).

### 2.2. Second Step: Direct Composite Restorations of Deep Carious Lesions

**Figure 4:**
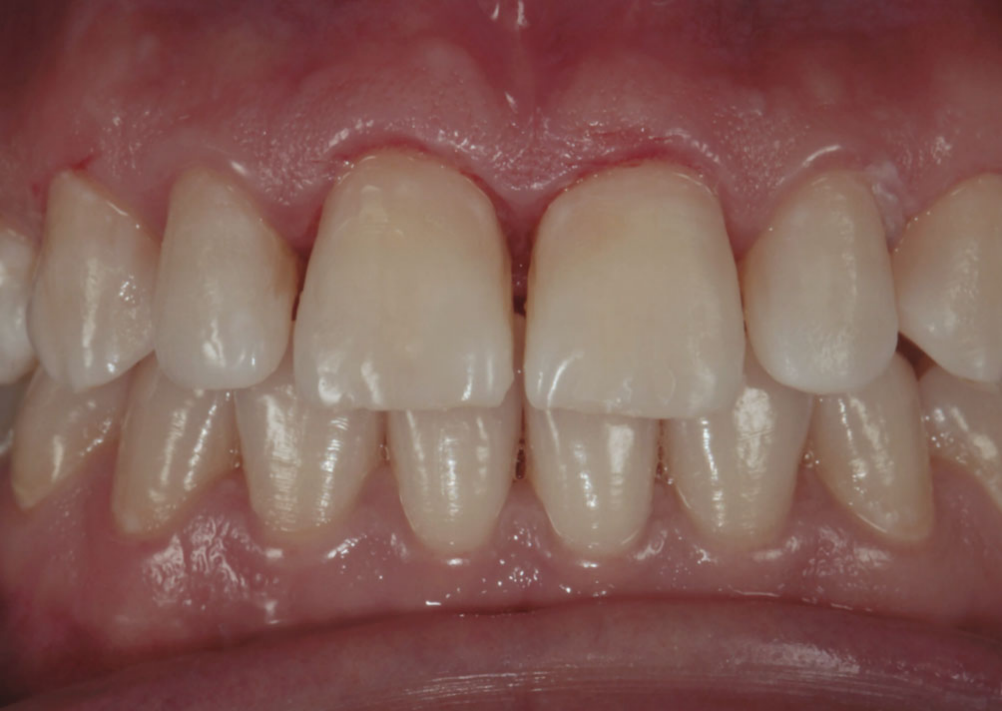
Final result just after rubber dam removal.

**Figure 5:**
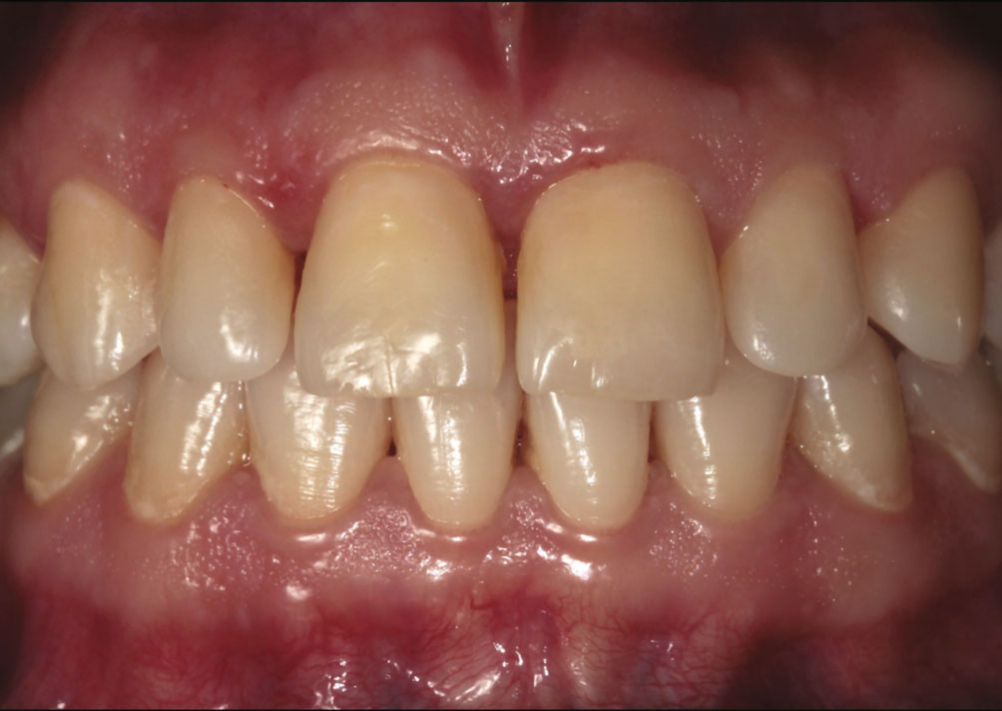
One-year recall.

**Figure 6:**
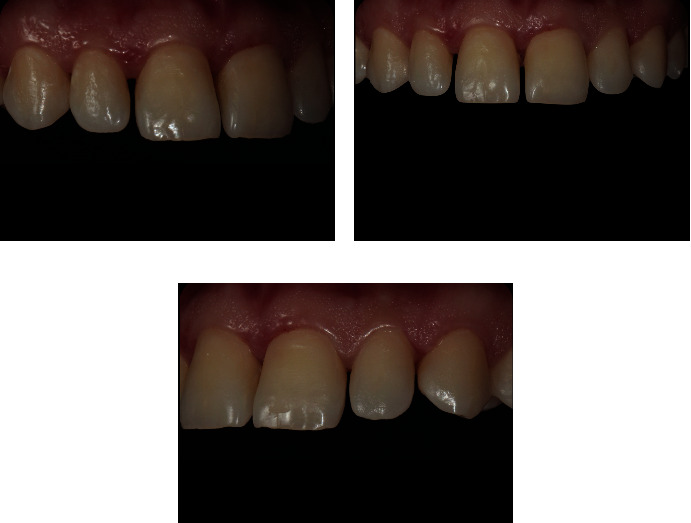
One year recall photographs with different angles. (a) Right angle, (b) Front angle, (c) Left angle.

After completion of the resin infiltration process, the restoration of the cavitated lesions was completed as follows. A universal adhesive (Prime&Bond Universal™, Dentsply-Sirona, Konstanz, Germany) and an A2 shade universal nano hybrid-composite with pre-polymerized fillers (Ceram.x^®^ SphereTEC™, Dentsply-Sirona) were used for the direct restorations. The lesions to be treated were etched, air-dried, and bonded according to the standard protocol of etch and rinse technique. Layering was carried out by using a composite brush and wetting resin (Composite Wetting Resin, Ultradent Products Inc., South Jordan, UT, USA). Finally, finishing and polishing procedures were done with abrasive finishing discs (Sof-Lex™, 3M ESPE) and a two-step finishing and polishing system (Enhance™PoGo™, Dentsply-Sirona), respectively. The final result immediately after the removal of the rubber dam and 1-year control revisit is shown in Figures 4, 5, and 6.

## 3. Discussion

An opaque white spot lesion is visibly distinct from the surrounding sound enamel due to the differences in the refractive index (RI) [3]. These lesions may compromise the smile esthetics if present in the esthetic zone, with the potential to progress to cavitated lesions in high caries-risk patients, especially when not managed adequately [11]. The principle of masking enamel lesions by resin infiltration is based on changes in light scattering within the lesions. Sound enamel has a RI of 1.62. The micro-porosities of enamel caries lesions are filled with either a watery medium (RI 1.33) or air (RI 1.0). The difference in refractive indices between the enamel crystals and the medium inside the porosities causes light scattering that results in an off-white, opaque appearance of these lesions, especially when they are air-dried [12]. The micro-porosities of infiltrated lesions are filled with resin (RI 1.46) that in contrast to the watery medium, cannot evaporate. As a result, the difference in refractive indices between porosities and enamel becomes negligible, leading to a seamless appearance of the lesions with the surrounding sound enamel [13].

Relative to conventional restorative techniques, caries infiltration is less invasive, requires only a negligible tooth substance to be sacrificed by etching and polishing, and represents a relatively fast treatment option for masking buccal non-cavitated caries lesions [14]. Similar to our study, Wierichs et al. [7] reported that the resin infiltration method continued its success in masking white spot lesions even after 6 years in their recent research. One of the reasons for this long success may be that the resin infiltration protects the initial lesions against acid attacks. Many studies claim that resin-infiltrated enamel is more resistant to acid attacks, and therefore to the development of caries lesions [15, 16].

On the other hand, Almansouri et al. [17] reported that resin infiltration application would not provide a greater benefit than other protective applications. Still, the vast majority stand behind the protective properties of resin infiltration. The overall success of the resin infiltration approach, including its masking effect, depends on the ability of the resin to infiltrate into porous spaces under an intact surface layer [18]. If cavitation has not occurred in the initial lesion, and the resin infiltration is performed as specified by the manufacturer, treatment will be successful. However, if cavitation has occurred, the current resin infiltration approach will be insufficient in the treatment.

In such cases, common practice is the replacement of the lost tissue by using resin composite materials with appropriate shades. In addition to their preserving feature due to little to no preparation required, resin composites also provide excellent esthetics in the anterior region [19]. It is known that the application of resin infiltration increases the bond strength between the white spot lesions and the resin composite [20]. Therefore, it can be said that it is a rational method to support resin composites in places where the resin infiltration method is insufficient.

## 4. Conclusion

In this case report, the application of the resin infiltration technique for the treatment of non-cavitated lesions before composite restoration of cavitated lesions enabled the preservation of tooth structures as much as possible, by eliminating the need for preparation of the tooth. Today, the importance of protecting dental tissue is well-recognized among dental practitioners. With this understanding, the described technique represents a useful option for dentists, whereby a combination of different treatment methods yields the most conservative form of treatment possible in cases where cavitated and non-cavitated lesions are present together.

## Data Availability

The data used to support the findings of this study are included within the article.

## References

[1] Winston A. E., Bhaskar S. N. (1998). Caries Prevention In The 21st Century. *The Journal of the American Dental Association*.

[2] Holmen L., Thylstrup A., Ogaard B., Kragh F. (1985). A polarized light microscopic study of progressive stages of enamel caries in vivo. *Caries Research*.

[3] Yuan H., Li J., Chen L., Cheng L., Cannon R. D., Mei L. (2014). Esthetic comparison of white-spot lesion treatment modalities using spectrometry and fluorescence. *The Angle Orthodontist*.

[4] Theodory T. G., Kolker J. L., Vargas M. A., Maia R. R., Dawson D. V. (2019). Masking and penetration ability of various sealants and icon in artificial initial caries lesions in vitro. *The Journal of Adhesive Dentistry*.

[5] Bailey D. L., Adams G. G., Tsao C. E. (2009). Regression of post-orthodontic lesions by a remineralizing cream. *Journal of Dental Research*.

[6] Bourouni S., Dritsas K., Kloukos D., Wierichs R. J. (2021). Efficacy of resin infiltration to mask post-orthodontic or non-post-orthodontic white spot lesions or fluorosis—a systematic review and meta-analysis. *Clinical Oral Investigations*.

[7] Wierichs R. J., Langer F., Kobbe C. (2023). Aesthetic caries infiltration—long-term masking efficacy after 6 years. *Journal of Dentistry*.

[8] Wierichs R. J., Abou-Ayash B., Kobbe C. (2023). Evaluation of the masking efficacy of caries infiltration in post-orthodontic initial caries lesions: 1-year follow-up. *Clinical Oral Investigations*.

[9] Sadıkoğlu İ. S. (2020). White spot lesions: recent detection and treatment methods. *Cyprus Journal of Medical Sciences*.

[10] Kielbassa A. M., Muller J., Gernhardt C. R. (2009). Closing the gap between oral hygiene and minimally invasive dentistry: a review on the resin infiltration technique of incipient (proximal) enamel lesions. *Quintessence International*.

[11] Araujo G., Naufel F., Alonso R., Lima D., Puppin-Rontani R. (2015). Influence of staining solution and bleaching on color stability of resin used for caries infiltration. *Operative Dentistry*.

[12] Kidd E. A., Fejerskov O. (2004). What constitutes dental caries? Histopathology of carious enamel and dentin related to the action of cariogenic biofilms. *Journal of Dental Research*.

[13] Rizzo N., da Cunha L. F., Sotelo B. V., Gonzaga C. C., Correr G. M., Gaião U. (2019). Esthetic rehabilitation with direct composite resin in a patient with amelogenesis imperfecta: a 2-year follow-up. *Case Reports in Dentistry*.

[14] Paris S., Meyer-Lueckel H. (2009). Masking of labial enamel white spot lesions by resin infiltration—a clinical report. *Quintessence International*.

[15] Enan E. T., Aref N. S., Hammad S. M. (2019). Resistance of resin-infiltrated enamel to surface changes in response to acidic challenge. *Journal of Esthetic and Restorative Dentistry*.

[16] Neres É. Y., Moda M. D., Chiba E. K., Briso A., Pessan J. P., Fagundes T. C. (2017). Microhardness and roughness of infiltrated white spot lesions submitted to different challenges. *Operative Dentistry*.

[17] Almansouri N., Bakry A. S., Abbassy M. A., Linjawi A. I., Hassan A. H. (2023). Evaluation of resin infiltration, fluoride and the biomimetic mineralization of CPP-ACP in protecting enamel after orthodontic inter-proximal enamel reduction. *Biomimetics*.

[18] Sadikoglu I. S., Arici M., Kemaloglu H., Turkun M., Caymaz M. G. (2022). Can the hydrogel form of sodium ascorbate be used to reverse compromised resin infiltrant penetration after bleaching?. *Nigerian Journal of Clinical Practice*.

[19] de Souza J. F., Fragelli C. M. B., Paschoal M. A. B. (2014). Noninvasive and multidisciplinary approach to the functional and esthetic rehabilitation of amelogenesis imperfecta: a pediatric case report. *Case Reports in Dentistry*.

[20] Soveral M., Machado V., Botelho J., Mendes J. J., Manso C. (2021). Effect of resin infiltration on enamel: a systematic review and meta-analysis. *Journal of Functional Biomaterials*.

